# Myeloid Lineage Ablation of *Phlpp1* Regulates M-CSF Signaling and Tempers Bone Resorption in Female Mice

**DOI:** 10.3390/ijms22189702

**Published:** 2021-09-08

**Authors:** Ismael Y. Karkache, Jeyaram R. Damodaran, David H. H. Molstad, Kim C. Mansky, Elizabeth W. Bradley

**Affiliations:** 1 Department of Orthopedics, School of Medicine, University of Minnesota, Minneapolis, MN 55455, USA; karka010@umn.edu (I.Y.K.); damod015@umn.edu (J.R.D.); molst031@umn.edu (D.H.H.M.); 2 Division of Orthodontics, Department of Developmental and Surgical Services, Institute for Virology, School of Dentistry, University of Minnesota, Minneapolis, MN 55455, USA; kmansky@umn.edu; 3 Stem Cell Institute, University of Minnesota, Minneapolis, MN 55455, USA

**Keywords:** Protein Kinase C (PKC), Akt, MEK, ERK, PH domain, Ras-association domain, PDZ domain, osteoclast, osteoporosis, sexual dimorphism, bone mass

## Abstract

Prior work demonstrated that Phlpp1 deficiency alters trabecular bone mass and enhances M-CSF responsiveness, but the cell types and requirement of Phlpp1 for this effect were unclear. To understand the function of Phlpp1 within myeloid lineage cells, we crossed *Phlpp1* floxed mice with mice harboring LysM-Cre. Micro-computed tomography of the distal femur of 12-week-old mice revealed a 30% increase in bone volume per total volume of *Phlpp1* female conditional knockouts, but we did not observe significant changes within male Phlpp1 cKO_LysM_ mice. Bone histomorphmetry of the proximal tibia further revealed that Phlpp1 cKO_LysM_ females exhibited elevated osteoclast numbers, but conversely had reduced levels of serum markers of bone resorption as compared to littermate controls. Osteoblast number and serum markers of bone formation were unchanged. In vitro assays confirmed that *Phlpp1* ablation enhanced osteoclast number and area, but limited bone resorption. Additionally, reconstitution with exogenous Phlpp1 suppressed osteoclast numbers. Dose response assays demonstrated that *Phlpp1^−/−^* cells are more responsive to M-CSF, but reconstitution with Phlpp1 abrogated this effect. Furthermore, small molecule-mediated Phlpp inhibition enhanced osteoclast numbers and size. Enhanced phosphorylation of Phlpp substrates—including Akt, ERK1/2, and PKCζ—accompanied these observations. In contrast, actin cytoskeleton disruption occurred within Phlpp inhibitor treated osteoclasts. Moreover, Phlpp inhibition reduced resorption of cells cultured on bovine bone slices in vitro. Our results demonstrate that *Phlpp1* deficiency within myeloid lineage cells enhances bone mass by limiting bone resorption while leaving osteoclast numbers intact; moreover, we show that Phlpp1 represses osteoclastogenesis and controls responses to M-CSF.

## 1. Introduction

Enhanced bone resorption is associated with conditions such as osteoporosis, cancer-associated bone loss, rheumatoid arthritis, and periodontitis. Bone resorption is accomplished by osteoclasts, the large, multinucleated cells that line bone surfaces. Osteoclasts arise via iterative fusion of myeloid progenitor cells and form within bone. Because disruptions of osteoclast differentiation and/or activity impact bone resorption, a better understanding of osteoclast biology will help design strategies to limit bone loss.

PH Domain and Leucine Rich Repeat Protein Phosphatase 1 (Phlpp1, Phlpp, Scop, Plekhe1, Ppm3a) is a serine/threonine protein phosphatase. Phlpp1 and its related isoform, Phlpp2, are metal-dependent type 2C protein phosphatases [1,2] that are insensitive to traditional phosphatase inhibitors [1]. Phlpp1 is broadly expressed, but its levels are controlled by numerous cellular mechanisms, and decline with age in women [1,2,3]. Our prior work also demonstrates that Phlpp1 levels are increased by estradiol in vitro [4].

PHLPP1 dephosphorylates and inactivates several kinases that regulate osteoclastogenesis and bone resorption including Akt, a kinase that is well established in promoting osteoclast differentiation, survival, and activity [5]. PHLPP1 catalyzes the dephosphorylation of AktS473, the site within the activation loop that is required for maximal Akt activity [2,6]. In addition to Akt, numerous PKC isoforms that promote osteoclast function are also inactivated and destabilized by PHLPP1-mediated dephosphorylation [2,7,8,9,10,11]. PHLPP1 also targets the pro-apoptotic Mst1 to limit cell survival and the ribosomal kinase p70 S6K to control the rate of CAP-dependent translation [9,11]. The activity of the Ras family small GTPases is attenuated by PHLPP1; thus, inhibiting MAPK signaling [7,9]. 

*Phlpp1* germline knockout mice (e.g., *Phlpp1^−/−^* mice) are viable, but show enhanced growth factor responsiveness and cellular proliferation [1]. Phlpp1 functions as a tumor suppressor and is frequently deleted in various cancers, including prostate cancers. In model systems, Phlpp1 inhibits tumor progression and cancer cell growth [1,2]. Our group previously showed that *Phlpp1* germline deficiency decreased body size, long bone length and bone mass [12]. In contrast, ablation of *Phlpp1* within Ctsk-Cre expressing cells enhanced bone mass [13], but the cell types responsible for this phenotype were unclear due to non-specific expression of the Ctsk-Cre transgene [14,15,16,17]. This study examines the effect of *Phlpp1* deletion within myeloid lineage cells on bone mass and osteoclastogenesis. 

## 2. Results

### 2.1. Deletion of Phlpp1 in LysM-Expressing Cells Enhances Bone Mass in Females

**Figure 1:**
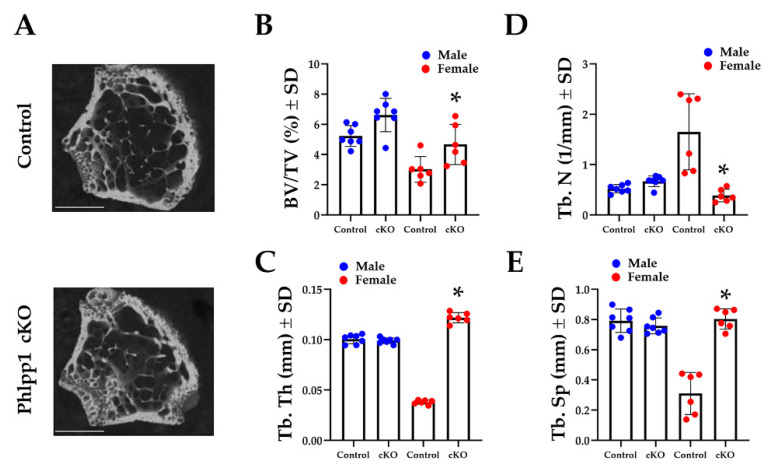
Conditional deletion of *Phlpp1* increases bone mass of female mice. *Phlpp1* cKO_LysM_ mice and their littermate controls were aged to 12 weeks and micro-CT was performed at the distal femur. (**A**) Representative images from female mice. Scale bar is 1 mm. (**B**) Bone volume per total volume (BV/TV), * *p* < 0.05. (**C**) Trabecular thickness (Tb. Th), * *p* < 0.05. (**D**) Trabecular number (Tb. N), * *p* < 0.05. (**E**) Trabecular spacing (Tb. Sp), * *p* < 0.05.

Our prior work demonstrated that germline deletion of *Phlpp1* limited acquisition of peak bone mass [12], but conditional deficiency within Ctsk-Cre expressing cells enhanced bone mass [13]. To determine how deletion of *Phlpp1* within myeloid progenitor cells affected bone mass, we crossed *Phlpp1* floxed [13] mice with mice expressing Lyz2-driven Cre (e.g., LysM-Cre) [18]. *Phlpp1* cKO_LysM_ and control littermates were aged to 12-weeks and femora and tibiae were collected. Micro-CT analyses revealed a 30 percent increase in trabecular BV/TV of *Phlpp1* cKO_LysM_ females within the distal femur (Figure 1A). This was accompanied by increased trabecular thickness (Figure 1B). In contrast, trabecular number was decreased, but trabecular spacing was elevated (Figure 1C,D). No significant trabecular bone changes were noted of *Phlpp1* cKO_LysM_ male mice at this age (Figure 1). No change in cortical bone at the femoral midshaft was noted of either males or females.

**Figure 2:**
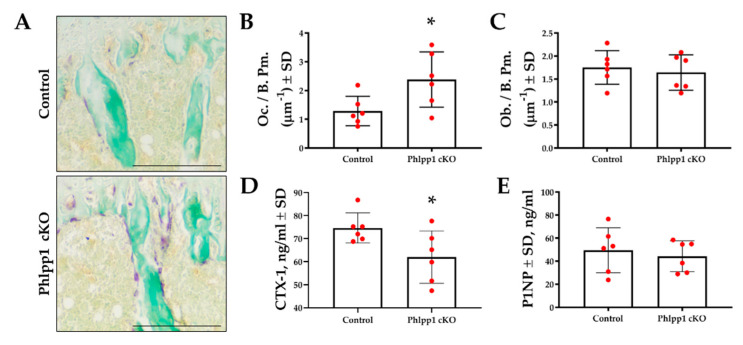
Conditional deletion of *Phlpp1* increases osteoclast number, but reduces bone resorption. Female *Phlpp1* cKO_LysM_ mice and control littermates were aged to 12 weeks and paraffin sections were collected. TRAP staining was performed (**A**) and the number of osteoclasts per bone perimeter (**B**) was determined. Scale bar is 500 microns. * *p <* 0.05. Sections were Goldner’s trichrome stained and numbers of osteoblasts per bone perimeter were determined (**C**). CTX-1 (**D**) and P1NP (**E**) ELISAs were performed using serum collected from 12-week-old female mice. * *p* < 0.05.

Bone histomorphometry was performed using tibiae of 12-week-old *Phlpp1* cKO_LysM_ mice and their control littermates to assess osteoclast and osteoblast numbers. TRAP-Fast Green staining was performed (Figure 2A,B) and demonstrated increased osteoclast number per bone perimeter (Figure 2A,B) within the proximal tibiae of *Phlpp1* cKO_LysM_ females. No changes in osteoblasts per bone perimeter were observed (Figure 2C). Serum ELISAs also demonstrated a 33% decrease in CTX-1 (Figure 2D), but no change in P1NP levels (Figure 2E).

### 2.2. Conditional Deletion of Phlpp1 Enhances Osteoclastogenesis

**Figure 3:**
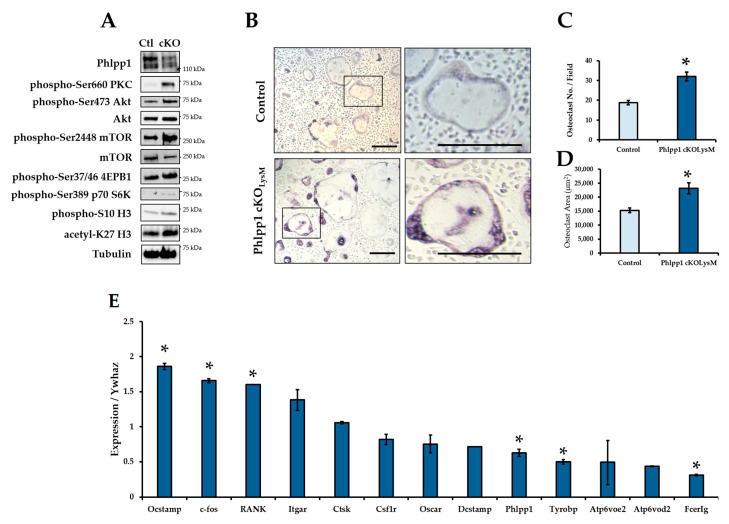
Conditional deletion of *Phlpp1* enhances ex vivo osteoclastogenesis. Osteoclasts were generated from bone marrow macrophages collected from 6–8-week-old *Phlpp1* cKO_LysM_ females or their sex-matched littermate controls. Samples were collected on day 4 and (**A**) Western blotting was performed. (**B**) TRAP staining of control or *Phlpp1* cKO_LysM_ cultures was performed. Insets in the left panels denote locations of higher magnification images shown in left panels. (**C**) Number of osteoclasts and (**D**) average osteoclast area per field were determined, * *p* < 0.05. (**E**) Expression of osteoclast phenotypic markers was assessed by qPCR, * *p* < 0.05. Scale bars are 100 microns.

Increased osteoclasts per bone surface were evident within 12-week-old *Phlpp1* cKO_LysM_ females. To further explore potential effects on osteoclast differentiation, ex vivo osteoclastogenesis assays were performed. Bone marrow macrophages were collected from *Phlpp1* cKO_LysM_ females and their sex-matched littermate controls and cultured in the presence of RANKL and M-CSF as previously described [13,19,20]. Diminished expression of Phlpp1 was confirmed of ex vivo osteoclasts derived from *Phlpp1* cKO_LysM_ mice (Figure 3A,E). Moreover, enhanced phosphorylation of Phlpp1 substrates was also observed (Figure 3A). Increased osteoclast number (59%) and area (66%) was also noted of *Phlpp1* cKO_LysM_ cultures (Figure 3B–D). This was accompanied by modest increases in OCstamp, c-fos and RANK expression (Figure 3E), but expression of Tyrobp and Fcerg was diminished (Figure 3E). No significant change in Itgar, Ctsk, Csf1r, Oscar, DCstamp, or Atp6voe2 was observed (Figure 3E). 

### 2.3. Phlpp1 Represses Ex Vivo Osteoclastogenesis

**Figure 4:**
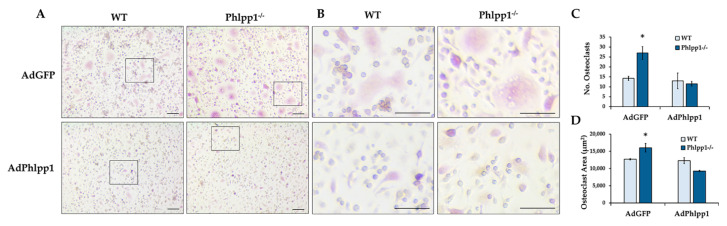
Phlpp1 Represses ex vivo Osteoclastogenesis. Osteoclasts were generated from bone marrow macrophages collected from 6–8-week-old *Phlpp1^−/−^* females or their sex-matched WT controls. Cultures were infected with each indicated adenovirus (MOI = 10) on day 0 and samples were collected on day 4. (**A**,**B**) TRAP staining of *Phlpp1^−/−^* or WT cultures. Insets in panel (**A**) denote locations of higher power images shown in (**B**). (**C**) Number of osteoclasts and (**D**) average osteoclast area per field were determined, *: *p* < 0.05 as compared to WT, AdGFP samples. Scale bars are 100 microns.

*Phlpp1* conditional deletion in LysM Cre-expressing cells enhanced ex vivo osteoclastogenesis. To determine if this could be restored by exogenous Phlpp1 reconstitution, we infected *Phlpp1^−/−^* osteoclast cultures with an adenovirus expressing Phlpp1. Bone marrow macrophages were collected from *Phlpp1^−/−^* females or their sex-matched littermates. Cells were infected with each indicated adenovirus on day 0 of culture. On day 4, cells were TRAP stained and the number and area of osteoclasts was evaluated (Figure 4). As previously reported [13], deletion of *Phlpp1* enhanced osteoclast number and area (Figure 4A–C). Reconstitution of Phlpp1 to endogenous levels suppressed osteoclastogenesis of control cultures and restored osteoclast number and area of *Phlpp1^−/−^* cultures (Figure 4A–C).

### 2.4. Reconstitution of Phlpp1 Restores M-CSF Responsiveness

**Figure 5:**
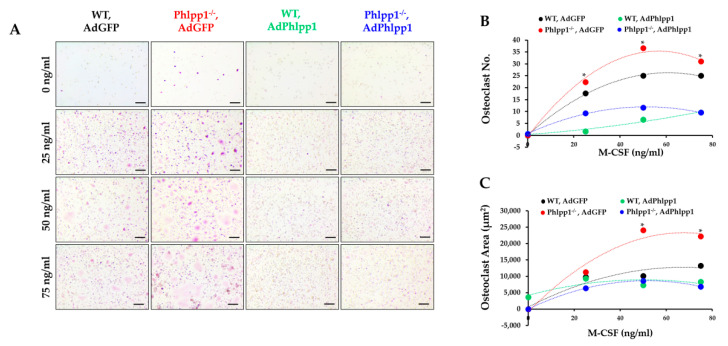
Phlpp1 attenuates responses to M-CSF. Osteoclasts were generated from bone marrow macrophages collected from 6–8-week-old *Phlpp1^−/−^* females or their sex-matched WT controls. Cultures were infected with each indicated adenovirus (MOI = 10) on day 0 and samples were collected on day 4. (**A**) TRAP staining of cultures was performed and the (**B**) number of osteoclasts and (**C**) average osteoclast area per field was determined, * *p* < 0.05 Scale bars are 100 microns.

Our prior work demonstrated that germline Phlpp1 deletion increased osteoclast responsiveness to M-CSF [13]. To demonstrate that Phlpp1 dampens M-CSF-mediated signaling, *Phlpp1^−/−^* or control littermate osteoclast cultures were infected with AdPhlpp1 or AdGFP and placed in ex vivo osteoclastogenesis assays with increasing M-CSF concentrations as shown (Figure 5). We confirmed that germline deletion of *Phlpp1^−/−^* enhances osteoclast number and area (Figure 5A–C). Overexpression of Phlpp1 in control osteoclast cultures repressed osteoclast number and area (Figure 5A–C). In addition, reconstitution of Phlpp1 within *Phlpp1^−/−^* cultures blunted osteoclastogenesis at all M-CSF concentrations tested (Figure 5A–C). 

### 2.5. Phlpp Inhibition Reduces Bone Resorption In Vitro

**Figure 6:**
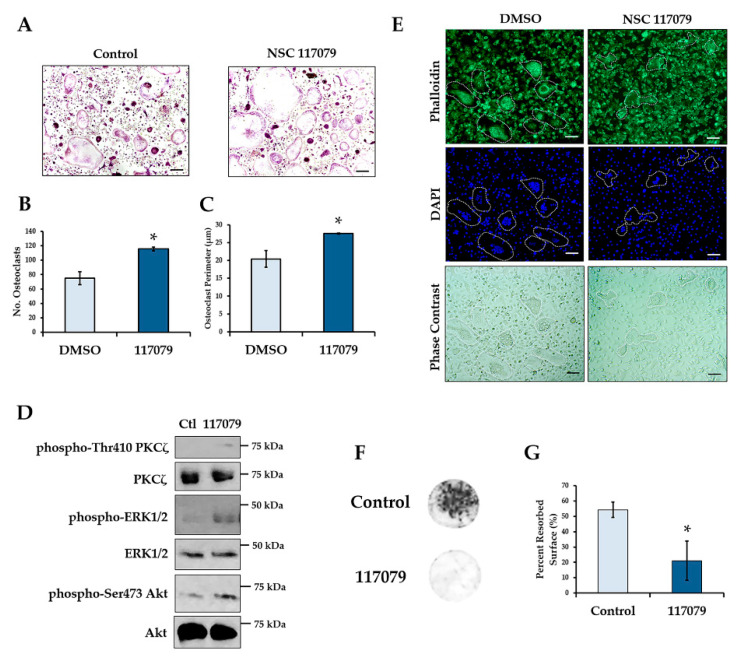
Phlpp inhibition reduces bone resorption ex vivo. Osteoclast progenitors were collected from 6–8-week-old C57Bl/6 females and cultured in the presence of the Phlpp inhibitor NSC 117079 in osteoclastogenic conditions. (**A**) TRAP staining was performed and the (**B**) number of osteoclasts and (**C**) average osteoclast area per field was determined, * *p* < 0.05. (**D**) Day 4 osteoclasts were stained with phalloidin and DAPI and bright field images were collected. (**E**) Western blotting was performed. (**F**,**G**) Osteoclasts were cultured on bovine bone disks in the presence of the Phlpp inhibitor NSC 117079 or control for 10 days. (**F**) Toluidine blue staining of the bone disks was performed, and (**G**) the average percent resorbed surface was determined. Scale bars are 100 microns.

Since conditional deletion of *Phlpp1* enhanced bone mass and reduced serum CTX-1 levels in vivo, we determined if small molecule-mediated inhibition of Phlpp isoforms limited bone resorption in vitro. Osteoclasts were generated from 4–6-week-old female C57Bl/6 mice and cultured in the presence of 5 μM NSC 117079 on day 0. Phlpp inhibition enhanced osteoclast numbers and size (Figure 6A–C). These observations were accompanied by enhanced phosphorylation of Phlpp substrates, including Akt, ERK1/2 and PKCζ (Figure 6D and Figure S1). In contrast, the actin cytoskeleton of osteoclasts was disrupted as compared to control treated cells (Figure 6E). Moreover, when cells were cultured on bovine bone slices for 10 days, Phlpp inhibition reduced bone resorption in vitro (Figure 6F,G).

## 3. Discussion

**Figure 7:**
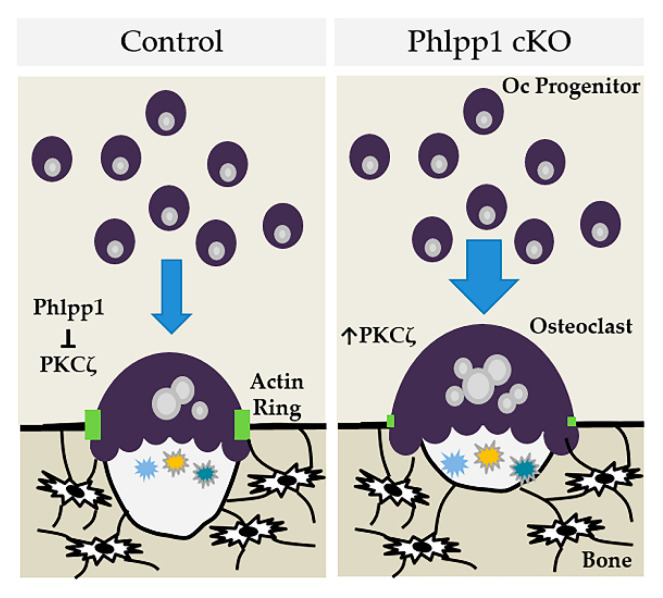
Model depicting how *Phlpp1* ablation may affect osteoclastogenesis and bone mass in female mice.

In this manuscript we demonstrate that conditional deletion of *Phlpp1* using LysM-driven-Cre results in enhanced bone mass within the distal femur of 12-week-old female mice, but not males. This phenotype is characterized by enhanced osteoclast numbers in vivo, but reduced serum CTX-1 levels. These results suggest that enhanced bone mass is due to reduced bone resorption. We show that enhanced osteoclastogenesis and M-CSF responsiveness can be restored by Phlpp1 reconstitution. We also demonstrate that elevated PKC signaling, in particular PKCζ, may mediate the Phlpp1-dependent changes in bone resorption (depicted in Figure 7). 

We previously showed that *Phlpp1* null mice have reductions in bone mass, but it was unclear if this effect was due to skeletal lineage cells or systemic factors [21]. In a subsequent manuscript, we demonstrated that *Phlpp1* deletion within Ctsk-expressing cells enhanced bone mass of female mice, but these effects could be attributed to expression of Ctsk-Cre within mesenchymal lineage cells and/or osteoclasts [13]. To address these limitations, we crossed *Phlpp1* floxed mice with the LysM-Cre driver mice and observed increased bone mass of 12-week-old female mice. These data demonstrate that myeloid lineage ablation of *Phlpp1* enhances bone mass.

The LysM-Cre driver is active within mono-myeloid cells that give rise to various types of macrophages within the body, including bone resorbing osteoclasts, but also osteomacs [22]. While it is possible that *Phlpp1* ablation within osteomacs has an anabolic effect bone formation, as these cells promote osteoblast mediated mineralization [23], makers of bone formation (e.g., P1NP) were unchanged in *Phlpp1* ablated animals. One limitation of this driver is that it can also induce genomic recombination of some alleles within neutrophils [24]. 

As previously reported and well documented within the literature, we observed that control male mice exhibit higher bone mass than their female littermates; thus, reflecting human bone physiology [25]. Ablation of *Phlpp1* increased trabecular bone mass of 12-week-old female *Phlpp1* cKO_LysM_ mice, but not males, suggesting that Phlpp1 has sexually dimorphic effects in regulating bone resorption. This possibility is further strengthened by the observation that *Phlpp1* levels decline with age in women, but are restored by short-term estrogen therapy [3]. Our prior work also shows that Phlpp1 expression by osteoclast progenitors is increased by estradiol [4]. Moreover, we demonstrated that enhanced bone mass of *Phlpp1* cKO_Ctsk_ females was dependent on reproductive status [4]. Future experiments will be aimed at determining if estradiol mediates the effects of *Phlpp1* ablation in vivo. 

We observed increased bone mass within the trabecular compartment of female *Phlpp1* ablated mice, but no changes in cortical bone. There are several possibilities to explain these findings. Bone remodeling within the trabecular compartment is a coupled process, in which bone resorption links to bone formation during the reversal phase. Our prior published data show that *Phlpp1* ablation enhances coupling to bone formation [13]. This coupling does not occur within cortical bone. This is one possible explanation for the lack of a cortical bone phenotype. It may also be possible that *Phlpp1* cKO_LysM_ mice acquire a cortical bone phenotype with increasing age, when endosteal bone resorption outpaces periosteal apposition.

Phlpp1, because it reduces anabolic signaling, suppresses cellular proliferation and survival of a many different cell types. Increased osteoclastogenesis occurring with myeloid lineage *Phlpp1* ablation may be due to enhanced proliferation of osteoclast progenitors. Phlpp1 was recently shown to counteract STAT1-mediated inflammatory signaling of macrophages [26]. Since the JAT/STAT inhibitor ruxolitinib decreases the proliferation and migration of Cd11b^+^ cells (e.g., osteoclast progenitors) [27], *Phlpp1* ablation may likewise enhance osteoclast progenitor proliferation and/or migration leading to increased osteoclast numbers. 

Diminished osteoclastogenesis observed with forced Phlpp1 expression (e.g., AdPhlpp1) could reflect several possibilities. Decreased osteoclast numbers could be a result of a permanent lineage change. As our models system uses unfractionated bone marrow macrophages, which are a heterogeneous population of cells, differential effects within this population is possible. Phlpp1 also targets phosphorylation of Histone 3, leading to altered acetylation and chromatin compaction [28]. Altered cell fate is a possibility; however, we did not observe a change in the number of TRAP^+^ mononuclear cells. Fusion and fission of osteoclast lineage cells could likewise be altered; as we see disruptions to the actin cytoskeleton of mature osteoclasts, this is a possibility worth exploring in the future. Phlpp1 also dampens pro-survival signaling; thus, diminished osteoclast survival could also account for lowered osteoclast numbers observed with AdPhlpp1 transduction. 

We found that small molecule-mediated inhibition of Phlpp isoforms likewise enhanced osteoclastogenesis, but limited bone resorption ex vivo. NSC 117079 inhibits the activity of PHLPP1 and PHLPP2, but inhibition of PHLPP2 activity is greater [29]. PHLPP1 dephosphorylates Akt2 and Akt3 isoforms, while PHLPP2 inactivates Akt1 and Akt3 [1,2]. Given Akt1 is expressed by osteoclasts and the NSC 117079 is more effective at blocking Phlpp2 activity, one future direction of this work is to examine the role of PHLPP2 in controlling bone mass and to assess any functional redundancy with PHLPP1. 

To mitigate bone loss, therapies target one or more aspects of bone remodeling. While anti-resorptive therapies (e.g., bisphosphonates, denosumab) effectively limit bone resorption, they also decrease osteoclast number leading to disruptions in the reversal phase [30,31,32,33]. In turn, this results in blunted osteoblast numbers and bone formation that negatively impacts bone quality [30,31,32,33]. In contrast, Cathepsin K inhibitors limit bone resorption without attenuating osteoclast number. This enhances bone mass without reducing bone formation rate. While Cathepsin K inhibitors failed in clinical trials, these data demonstrate that targeting osteoclast activity without reducing numbers may be a therapeutic avenue to enhance bone mass [34,35,36,37]. Thus, therapies designed to limit osteoclast activity and/or restore coupling may enhance bone mass and retain bone quality. Overall, our data demonstrate that Phlpp1 suppresses osteoclastogenesis, but also plays positive role in establishing osteoclast polarity and bone resorption. Targeting Phlpp1 in committed osteoclasts may be a strategy to enhance bone mass in females, or could be used synergistally with current anti-resorbtive and/or anabolic treatments. 

## 4. Materials and Methods

### 4.1. Generation of Phlpp1 Germline and Conditional Knockout Mice

Mice harboring a floxed *Phlpp1* allele were crossed with mice expressing Cre recombinase driven by the Lyz2 promoter (LysM-Cre). We crossed Phlpp1^fl/fl^: Cre^−^ animals with Phlpp1^fl/+^: Cre^+^ animals to generate *Phlpp1* conditional knockout mice (referred to as *Phlpp1* cKO_LysM_) and were genotyped as previously described [13,18]. Generation and genotyping of *Phlpp1^−/−^* animals was performed as previously described [38]. All animals are fully backcrossed to the C57Bl/6 strain. Animals were housed in an accredited facility under a 12-h light/dark cycle and provided water and food ad libitum. All animal research was conducted according to guidelines provided by the National Institute of Health and the Institute of Laboratory Animal Resources, National Research Council. The University of Minnesota Institutional Animal Care and Use Committee approved all animal studies.

### 4.2. Micro-Computed Tomography

Femora from 12-week-old male and female *Phlpp1* cKO_LysM_ mice (*n* = 6 per sex) and their control littermates (Phlpp1^fl/fl^: Cre^−^, *n* = 4 or Phlpp1^fl/+^: Cre^−^ animals, *n* = 2 per sex) were isolated and fixed in 10% neutral buffered formalin for 48 h, then stored in 70% ethanol. Scanning was performed using a Scanco Viva40 micro-CT at 70 kV, 221 ms with a 10.5-μm voxel size. For trabecular measurements, a region of interest was defined at 10% of total femur length starting immediately proximal to the growth plate; samples were analyzed using a 220-threshold air filling correction.

### 4.3. Histology and Histomorphometry

Tibiae from 12-week-old male and female *Phlpp1* cKO_LysM_ mice (*n* = 6) and their control littermates (Phlpp1^fl/fl^: Cre^−^, *n* = 4 or Phlpp1^fl/+^: Cre^−^ animals, *n* = 2 per sex) were isolated and fixed in 10% neutral buffered formalin for 48 h, decalcified in 15% EDTA for 14 days, then stored in 70% ethanol. Tissues were paraffin embedded and 7-μm sections were collected and TRAP/Fast Green stained (Sigma, #387A-1KT) or Masson’s trichrome stained as previously described [13]. Standardized histomorphometry was performed [39]. 

### 4.4. Serum Markers of Bone Resorption and Formation

Serum was collected from 12-week-old *Phlpp1* cKO_LysM_ (*n* = 6) female mice and their control littermates (Phlpp1^fl/fl^: Cre^−^, *n* = 4 or Phlpp1^fl/+^: Cre^−^ animals, *n* = 2 per sex) and stored at −80 °C. Enzyme-linked immunosorbent assays (ELISA) for bone resorption (CTX-1) were performed in duplicate using 20 μL of serum from each mouse according to the manufacturer’s specifications (Ratlaps (CTX-1) #AC-06F1, Immunodiagnostics Systems). Bone formation was assessed using the ELISA for serum P1NP performed in duplicate using serum from each mouse as described by the manufacturer (Ratlaps (P1NP), #AC-33F1, Immunodiagnostics Systems, East Boldon, UK).

### 4.5. Osteoclastogenesis and Bone Resorption Assays

Bone marrow macrophages were collected from *Phlpp1* cKO_LysM_ mice or *Phlpp1^−/−^* mice and their control littermates as previously described [40]. Cells were cultured in phenol red-free α-MEM overnight in the presence of 35 ng/mL rM-CSF (#410-ML, R&D Systems, Minneapolis, MN, USA). Non-adherent cells were collected and cultured with 90 ng/mL rRANKL (#315-11, PreproTech, Rocky Hill, NJ, USA) and 35 ng/mL M-CSF at a density of 0.4x10^6^ cells/cm^2^ [40] and infected with each indicated adenovirus (MOI = 10). For dose–response assays using *Phlpp1^−/−^* cells, cells were exposed to increasing M-CSF concentrations and infected with adenoviral (Ad) GFP or AdPhlpp1 at an MOI of 10 as previously described [41,42]. On day 4, cells were either fixed or lysed on ice in a buffered SDS (0.1% glycerol, 0.01% SDS, 0.1 m Tris, pH 6.8) for further analyses. For enumeration of osteoclast number and size, cells were TRAP (#387A-1KT, Sigma-Aldrich, St. Louis, MO, USA) and DAPI (#H-1200-10, Vector Biolabs) stained. The number and size of TRAP^+^ cells with 3 or more nuclei was determined as previously described [13]. All experiments were repeated three times with independent biological replicates.

Bone marrow macrophages from C57Bl/6 females (*n* = 3) were also cultured in the presence of the small molecule Phlpp inhibitor NSC 117079 (5 μM, MedChemExpress, #HY-19819) starting on day 0 of osteoclastogenesis assays. Cells were fed on day 3 with osteoclastogenic medium containing NSC 117079 or control. On day 4, cells were TRAP stained. Bone marrow macrophages were also seeded onto bovine bone disks. Cells were cultured on bone slices for 10 days and feed with osteoclastogenic medium containing NSC 117079 or control every 3–4 days. On day 10, cells were lysed in 5% domestic bleach and bone slices were stained with toluidine blue as previously described [13,19].

### 4.6. Western Blotting

For Western blotting, cell lysates were collected in SDS sample buffer on ice. After determining total protein concentrations using the Bio-Rad D_C_ assay (Bio-Rad, Hercules, CA, USA), 20 μg of total proteins per sample was resolved by SDS-PAGE and transferred to PVDF membranes. Western blotting was performed with antibodies (1:2000 dilution) for PHLPP1 (#07-1342, Millipore, Burlington, MA, USA), phospho-Ser 660 PKC (#9371, Cell Signaling Technology, Danvers, MA, USA), phospho-Ser 410 PKCζ (#MA5-15060, Invitrogen), PKCζ (#9372, Cell Signaling Technology), phospho-Akt2 (#8599, Cell Signaling Technology), Akt (#4691, Cell Signaling Technology), phospho-Ser2448 mTOR (#5536, Cell Signaling Technology), mTOR (#2972, Cell Signaling Technology), phospho-Ser56 4E-BP1 (#9456, Cell Signaling Technology), phospho-Ser371 p70 S6K (#9204, Cell Signaling Technology), phospho-Thr389 p70 S6K (#9205, Cell Signaling Technology), phospho-Ser10 Histone 3 (#9701, Cell Signaling Technology), acetyl-K27 Histone 3 (#ab4729, Abcam, Cambridge, UK), Histone 3 (#ab176840, Abcam), Tubulin (E7, Developmental Hybridoma Bank), and corresponding secondary antibodies conjugated to horseradish peroxidase (HRP) (Cell Signaling Biotechnology). Protein detection was accomplished using the Supersignal West Femto Chemiluminescent Substrate (Pierce, Waltham, MA, USA). Experiments were repeated three times with biological replicates with representative data shown.

### 4.7. Statistics

GraphPad Prism 8 software (version 8.3.0, San Diego, CA, USA) was utilized for statistical analyses. Comparisons were made using a Student’s *t*-test or AVOVA as appropriate. Comparisons where *p* < 0.05 were considered statistically significant.

## Data Availability

All data are contained within this manuscript.

## Supplementary Materials

The following are available online at https://www.mdpi.com/article/10.3390/ijms22189702/s1.
