# Supplementary material for: Myeloid Lineage Ablation of Phlpp1 Regulates M-CSF Signaling and Tempers Bone Resorption in Female Mice
Source: Int J Mol Sci. 2021 Sep 8;22(18):9702. doi: 10.3390/ijms22189702 (PMC8468863; doi:10.3390/ijms22189702)
Supplement: Supplementary file 1 [file ijms-22-09702-s001.zip › Supplemental Information_08182021.pdf]

International Journal of *Molecular Sciences*

MDPI

Supplementary

Myeloid Lineage Ablation of *Phlpp1* Regulates M-CSF Signaling and Tempers Bone Resorption in Female Mice

Ismael Y. Karkache [1], Jeyaram R. Damodaran [1], David H. H. Molstad [1], Kim C. Mansky [2], and Elizabeth W. Bradley [1,3,*]

[1] Department of Orthopedics, School of Medicine, University of Minnesota, Minneapolis, MN 55455, USA; karka010@umn.edu (I.Y.K.); damod015@umn.edu (J.R.D.); molst031@umn.edu (D.H.H.M.)
[2] Division of Orthodontics, Department of Developmental and Surgical Services, Institute for Virology, School of Dentistry; University of Minnesota, Minneapolis, MN 55455, USA, kmansky@umn.edu
[3] Stem Cell Institute, University of Minnesota, Minneapolis, MN 55455, USA
* Correspondence: ebradle1@umn.edu

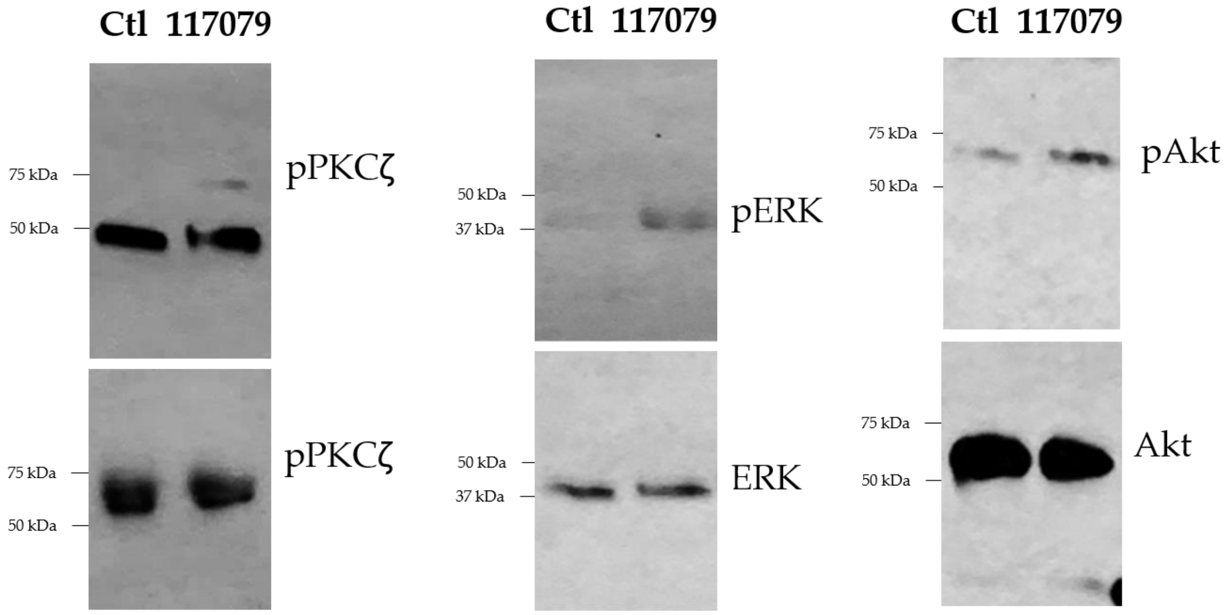

Figure S1. Uncropped blots from Figure 6D.
